# 648 Hand Burns in Low and Middle-Income Countries: Insights from the Global Burn Registry

**DOI:** 10.1093/jbcr/iraf019.277

**Published:** 2025-04-01

**Authors:** Daniel Najafali, Hilary Liu, Saeid Rezaei, José Arellano, Erik Reiche, Quincy Tran, Victor Stams, Francesco Egro

**Affiliations:** Carle Illinois College of Medicine; University of Pittsburgh Medical Center; University of Pittsburgh Medical Center; University of Pittsburgh Medical Center; University of Pittsburgh Medical Center; University of Maryland School of Medicine; Carle Foundation Hospital; University of Pittsburgh Medical Center

## Abstract

**Introduction:**

Hand burn injuries have been well described in high-income countries, but less evidence has been established for low- and middle-income countries (LMIC). Burns to the hand are a significant cause of functional impairment and disability, particularly in LMICs, where access to specialized burn care and rehabilitation is limited. There is a need to provide an overview of hand burn management and outcomes in LMICs. This study analyzes the treatment patterns and outcomes of hand burns in LMICs. We hypothesized that limited access to specialized burn care and surgical interventions in these regions leads to higher rates of physical impairment and mortality among hand burn patients.

**Methods:**

Hand burns were extracted from the WHO Global Burn Registry from inception to September 2024. Individuals from high-income countries were excluded. Descriptive analysis summarized patient demographics, burn characteristics, and hospital care variables. Multivariable logistic regression was performed to identify factors associated with the need for surgery and poorer outcomes in LMIC settings, including mortality and functional impairment at discharge for survivors.

**Results:**

There were 4,169 cases from LMICs with 2,121 (51%) hand burns. Patients with hand burns had a significantly higher median age at the time of burn injury (28 vs. 18 years, P< 0.001). Most patients with hand burns in LMICs were male (56%), had flame-based etiologies (67%), had median total body surface area (TBSA) burned of 30% (IQR 15-50%), and sustained a significantly higher proportion of inhalation injury (36%) compared to non-hand burn patients (10%, P< 0.001). Patients with hand burns sustained a significantly higher percentage of burns to the head and neck, trunk, arm, and lower extremities (P< 0.001). Physical impairment at discharge among survivors (non-hand: 8% vs. hand: 13%, P< 0.001) and mortality (non-hand: 16% vs. hand: 42%) was significantly higher (P< 0.001) among patients who sustained a burn to the hand. More patients without hand burns underwent surgery (44% vs. 36%, P< 0.001) and were discharged home (73% vs. 46%, P< 0.001). In multivariable analysis, hand burns were significantly associated with lower odds of surgical intervention (OR 0.86, 95%CI 0.74-0.99). When focusing on survivors, hand burns were significantly associated with higher odds of being discharged home with a physical impairment (OR 1.40, 95%CI 1.06-1.87), but did not confer an increased risk for mortality (P>0.05) when controlling for demographic and clinical features.

**Conclusions:**

Hand burns in LMICs had disproportionately greater physical impairment at discharge and higher mortality rates compared to non-hand burn injuries. The study underscores that hand burns in LMICs result in poor functional outcomes.

**Applicability of Research to Practice:**

Implementing interventions that focus on improving surgical capacity and post-burn rehabilitation in LMICs could lead to better outcomes for survivors of hand burns.

**Funding for the Study:**

N/A

